# The effects of heated humidification to nasopharynx on nasal resistance and breathing pattern

**DOI:** 10.1371/journal.pone.0210957

**Published:** 2019-02-06

**Authors:** Yukio Fujita, Motoo Yamauchi, Hiroki Uyama, Hideshi Oda, Michihito Igaki, Masanori Yoshikawa, Hiroshi Kimura

**Affiliations:** 1 Second Department of Internal Medicine (Department of Respiratory Medicine), Nara Medical University, Kashihara, Japan; 2 Personal Health Care Products Research Laboratories, Kao Corporation, Tokyo, Japan; 3 Department of Advanced Medicine for Pulmonary Circulation and Respiratory Failure, and Department of Pulmonary Medicine, Nippon Medical School Graduate School of Medicine, Tokyo, Japan; National Taiwan University Hospital, TAIWAN

## Abstract

**Background:**

Mouth breathing could induce not only dry throat and eventually upper respiratory tract infection, but also snoring and obstructive sleep apnea, while nasal breathing is protective against those problems. Thus, one may want to explore an approach to modify habitual mouth breathing as preferable to nasal breathing. The aim of this study was to investigate the physiological effects of our newly developed mask on facilitation of nasal breathing.

**Methods:**

Thirty seven healthy male volunteers were enrolled in a double blind, randomized, placebo-controlled crossover trial. Participants wore a newly developed heated humidification mask or non-heated-humidification mask (placebo) for 10-min each. Subjective feelings including dry nose, dry throat, nasal obstruction, ease to breathe, relaxation, calmness, and good feeling were asked before and after wearing each mask. In addition, the effects of masks on nasal resistance, breathing pattern, and heart rate variability were assessed.

**Results:**

Compared with the placebo mask, the heated humidification mask improved all components of subjective feelings except for ease to breathe; moreover, decreased nasal resistance and respiratory frequency accompanied a simultaneous increase in a surrogate maker for tidal volume. However, use of the heated humidification mask did not affect heart rate variability

**Conclusion:**

Adding heated humidification to the nasopharynx could modulate breathing patterns with improvement of subjective experience and objective nasal resistance.

## Introduction

Humans breathe primarily through nose, but some breathe through the mouth. Mouth breathing could induce dry throat, while nasal breathing has protective effects on dry throat since the nasal mucosa adds heat and humidification to inspired air during inspiration[1]. Dry throat and dry mouth are considered as risk factors for upper and lower respiratory tract infection[2], thus nasal breathing would be preferable to mouth breathing. Furthermore, mouth breathing enhances snoring and obstructive sleep apnea since genioglossus activity during nasal breathing has been reported to be higher compared to mouth breathing[3]. Taken together, changing breathing route, which is from mouth to nose, would provide many benefits for humans.

People, especially East Asians, use mouth coverings and nasal masks to prevent droplet/air infections, but those with nasal obstruction sometimes feel uncomfortable to breathe with the mask. One of the reasons might be lack of heat and humidification. It has been reported that local thermotherapy, such as in the facial eyelid region[4] and in the neck region[5], was useful to ameliorate mental stress and fatigue. Based on these background findings, our first attempt was to develop a new mask which can add heat and humidification, and then investigation was performed to identify whether the mask can improve nasal resistance and subjective feelings including relaxation, nasal symptoms, and so forth. Furthermore, additional evaluation was conducted to examine whether breathing pattern is modulated by our newly developed mask, with the assumption that the mask can change breathing route due to decreased nasal resistance.

## Materials and methods

### Participants

Thirty seven healthy male volunteers participated in this study. Subjects who were under medication for chronic lung, cardiac, and kidney diseases were excluded. All participants gave written informed consent, and this study is approved by the Ethical Advisory Committee at Nara Medical University (No. 809).

### Study protocol

**Fig 1:**
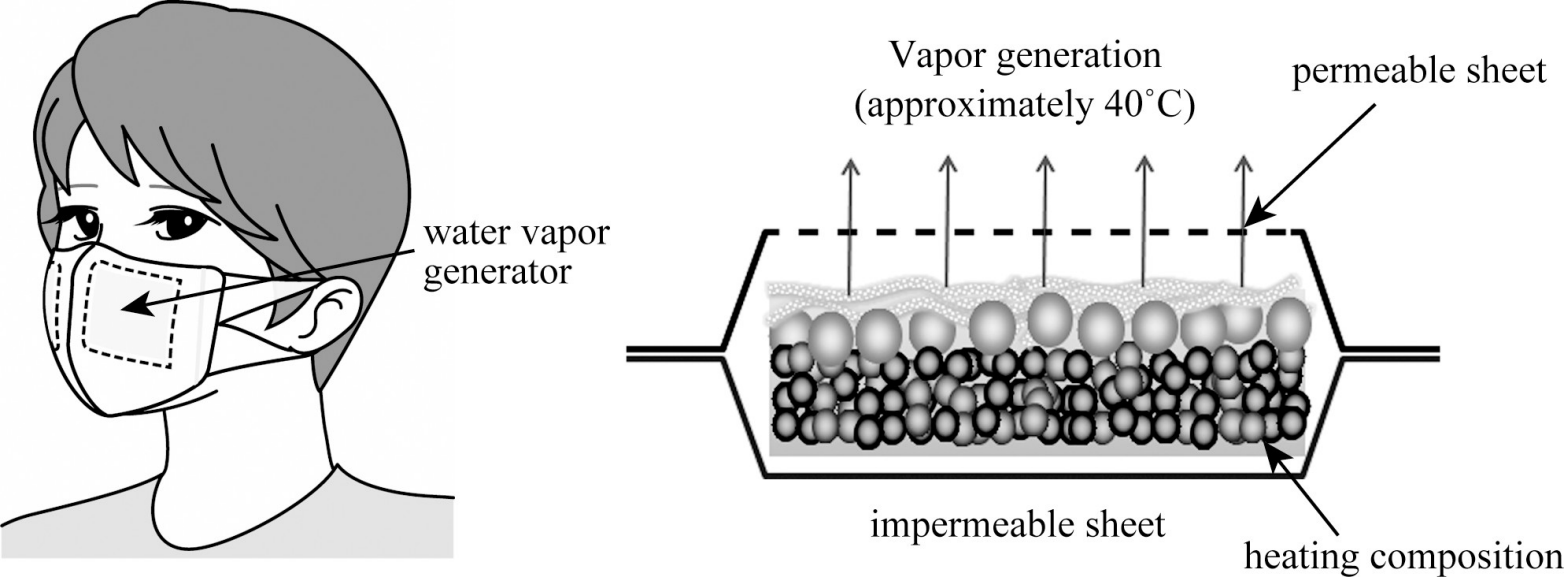
Structure of heated humidification mask. Heated humidification mask has a 3-dimensional structure. Heat-and-steam generator (HSG) attached on the top of the mask produces warm water vapor through the oxidation reaction of iron. The water contained in the heating component inside the HSG is generated as warm steam from the permeable sheet side. Skin temperature in the mask is kept at around 38–40˚C. HSG = heat-and-steam generator.

The study design was a double blind, randomized, placebo-controlled crossover trial. First, all participants were kept in the specific environment variable room, where experiments were performed, for 30 minutes in the daytime in order to acclimatize themselves to that environment. The setting of the temperature and the relative humidity in the room was constantly 20 degrees Celsius and 10% (absolute humidity of 1.7g/m^3^). Then, experiments were performed under the same settings after an acclimatization period. Participants wore a newly developed heated humidification mask for 10 to 20 minutes, which is produced by Kao Corporation (Tokyo, Japan), or non-heated-humidification mask (placebo). The heated humidification mask has a 3-dimensional structure. Specifically, the heat-and-steam generator (HSG) attached on the top of the mask produces warm water vapor through the oxidation reaction of iron. The HSG generates heat and steam via the following chemical reaction. Fe + 3/4O_2_ + 3/2H_2_O = > Fe(OH)_3_ + 402kcal/mol + steam. The water contained in the heating component inside the HSG is generated as warm steam is generated from the permeable sheet side. A 3-dimensional structure enables the ability to maintain the space inside the mask and to inhale generated water vapor effectively (Fig 1). The skin temperature in the mask is kept at around 38–40˚C. The placebo mask (non-heated-humidification mask) was similarly prepared without the capability of generating warm steam.

**Fig 2:**
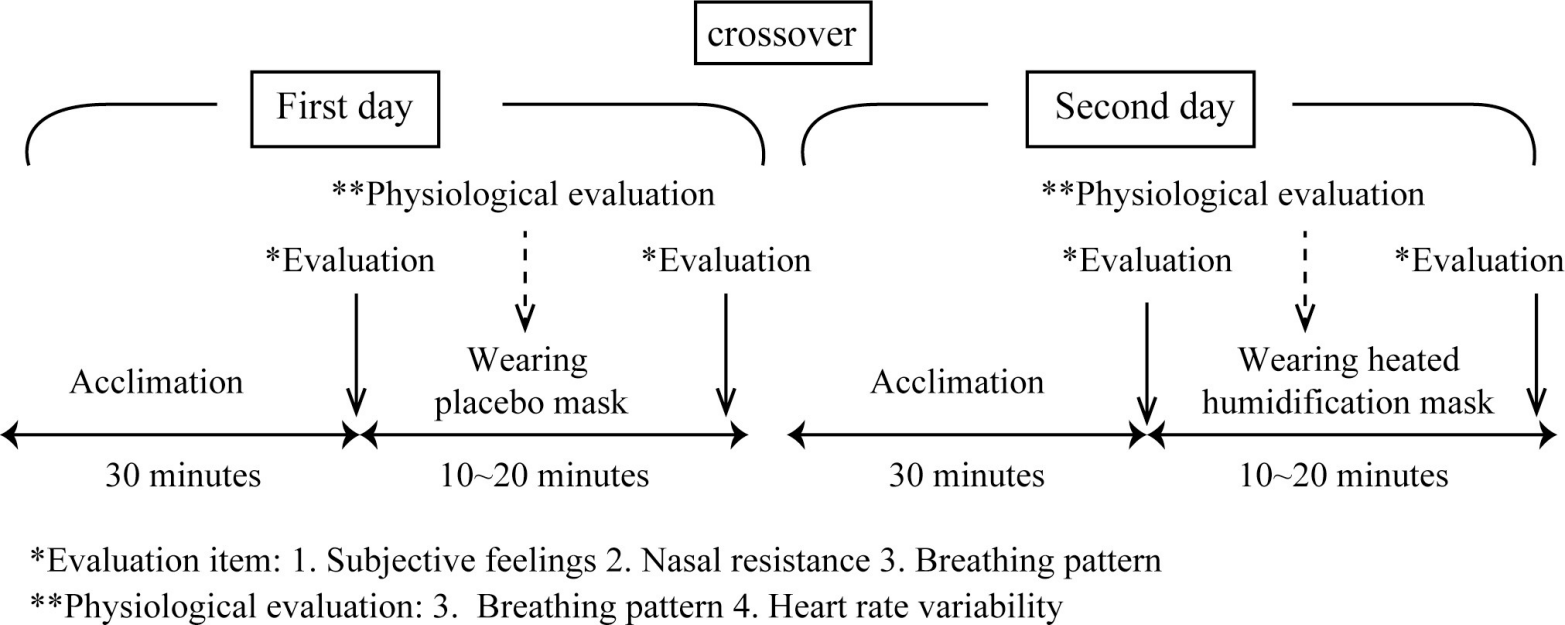
Study protocol. Subjective feelings and nasal resistance were compared before and after wearing placebo mask or heated humidification mask, and breathing pattern and heart rate variability during the period of wearing the mask were assessed.

Data collection was performed during the same time over two different days. Then, subjective feelings and nasal resistance were compared before and after wearing each mask, and breathing pattern and heart rate variability during the period of wearing the mask were assessed (Fig 2).

### Subjective feelings

Subjective feelings included dry nose, dry throat, nasal obstruction, ease to breathe, relaxation, calmness, and good feeling were assessed using Visual Analogue Scale (VAS), which is a measuring instrument for the documentation of symptoms. On the 100mm long horizontal line with verbal descriptions, participants mark the point on the line before and after wearing each mask. Then, the improvement of symptoms was evaluated as the absolute change in millimeters.

### Nasal resistance

Nasal resistance was measured with an anterior rhinomanometry (MPR-3100, Nihon-Kohden, Tokyo, Japan) [6, 7]. Briefly, left and right unilateral measurements were performed, and all nasal resistance measurements were calculated according to Ohm’s law. Total inspiratory nasal resistance at negative 100 Pa was calculated [8]. These procedures were repeated three times, and mean value of those was adopted as nasal resistance.

### Breathing pattern

Participants were given no information about what we expect and about the hypotheses; in addition, participants were given no instruction on how to breathe. Resting breathing during wakefulness was collected using nasal pressure sensor (LS-300, Fukuda Denshi, Tokyo, Japan) for approximately 20min of wearing mask in the seated position in the specific environment variable room we mentioned above. The sampling rate of respiratory signal acquisition was 12.5Hz. The details of this device have been described in our previous papers[9, 10]. Participants were requested to keep their eyes open. Six-minutes of artifact-free respiratory signal was extracted for the evaluation of breathing pattern. We did not perform biological calibration before data collection since the scope of this study was the comparison of the values within individual not between individual. Therefore, the term “surrogate marker for tidal volume” was used rather than “tidal volume” in the current study.

Breathing pattern was assessed by mean respiratory frequency and surrogate marker for tidal volume obtained from the calculations of breath-to-breath values.

### Heart rate variability

ECG signals were obtained from Holter system (LS-300, Fukuda Denshi, Tokyo, Japan) at a sampling frequency of 125Hz in 35 subjects during the period of wearing mask, which was approximately for 10-20min. Two subjects were excluded from the analysis because of poor ECG signals. In power spectral analysis of heart rate variability, the variance of low-frequency component (LF; 0.04–0.15Hz), high-frequency component (HF; 0.15–0.40Hz), and LF to HF ratio (LF/HF) were measured. It is reported that LF/HF reflects mainly sympathetic nervous system and HF reflects mainly parasympathetic nervous system[11].

### Statistical analysis

Continuous variables are presented as average ± standard deviation (SD). Comparison of each parameter between two groups was made by paired T-test. Differences with p<0.05 were considered significant. Statistical analysis was done with IBM SPSS Statistics 20 for Windows software (SPSS Inc., Chicago, IL, USA).

## Results

### Subject characteristics

The age and body mass index of all subjects were 37.6± 9.3 years old and 22.1± 2.0 kg/m^2^.

### Subjective feelings

Although the placebo mask improved only dry nose and dry throat as compared with those before wearing placebo mask, the heated humidification mask significantly improved all subjective feelings (Table 1). Moreover, as for the degree of the improvement in VAS score before and after wearing each mask, improvement of all components of subjective feelings except ease to breathe with heated humidification mask were significantly greater than with placebo mask (Table 2).

**Table 1:** Subjective feelings before and after wearing each mask in VAS score. Data are presented as mean ± SD, the unit is mm.

Evaluation item	placebo mask			heated humidification mask		
	before wearing mask	after wearing mask	p-value	before wearing mask	after wearing mask	p-value
Dry nose	43.9±27.2	33.6±24.2	<0.01	42.1±26.8	24.0±21.0	<0.01
Dry throat	43.6±25.1	35.6±25.1	<0.01	49.5±26.2	31.1±22.7	<0.01
Nasal obstruction	29.0±23.9	25.7±22.3	N.S.	34.7±26.0	19.5±17.3	<0.01
Ease to breathe	56.0±27.2	58.3±22.8	N.S.	58.0±23.2	66.0±22.2	<0.05
Relaxation	75.2±17.6	76.1±18.2	N.S.	69.7±19.0	78.0±13.3	<0.01
Calmness	77.0±18.8	76.4±16.7	N.S.	73.5±17.6	78.8±14.5	<0.01
Good feeling	68.5±16.9	69.1±16.1	N.S.	66.1±14.0	74.4±14.0	<0.01

**Table 2:** Improvements in subjective feeling in VAS score.

Evaluation item	placebo mask	heated humidification mask	p-value
Dry nose	+10.3 ± 15.1	+18.2 ± 17.9	<0.01
Dry throat	+8.0 ± 14.8	+18.3 ± 20.2	<0.01
Nasal obstruction	+3.2 ± 11.3	+15.2 ± 20.4	<0.01
Ease to breathe	+2.4 ± 21.0	+8.0 ± 21.5	N.S.
Relaxation	+0.9 ± 8.5	+8.3 ± 11.7	<0.01
Calmness	-0.6 ± 9.7	+5.3 ± 9.8	<0.01
Good feeling	+0.6 ± 10.7	+8.3 ± 8.9	<0.01

Data are presented as mean ± SD, the unit is mm.

The first + and–in each cell mean an improvement and a deterioration, respectively

### Nasal resistance

**Fig 3:**
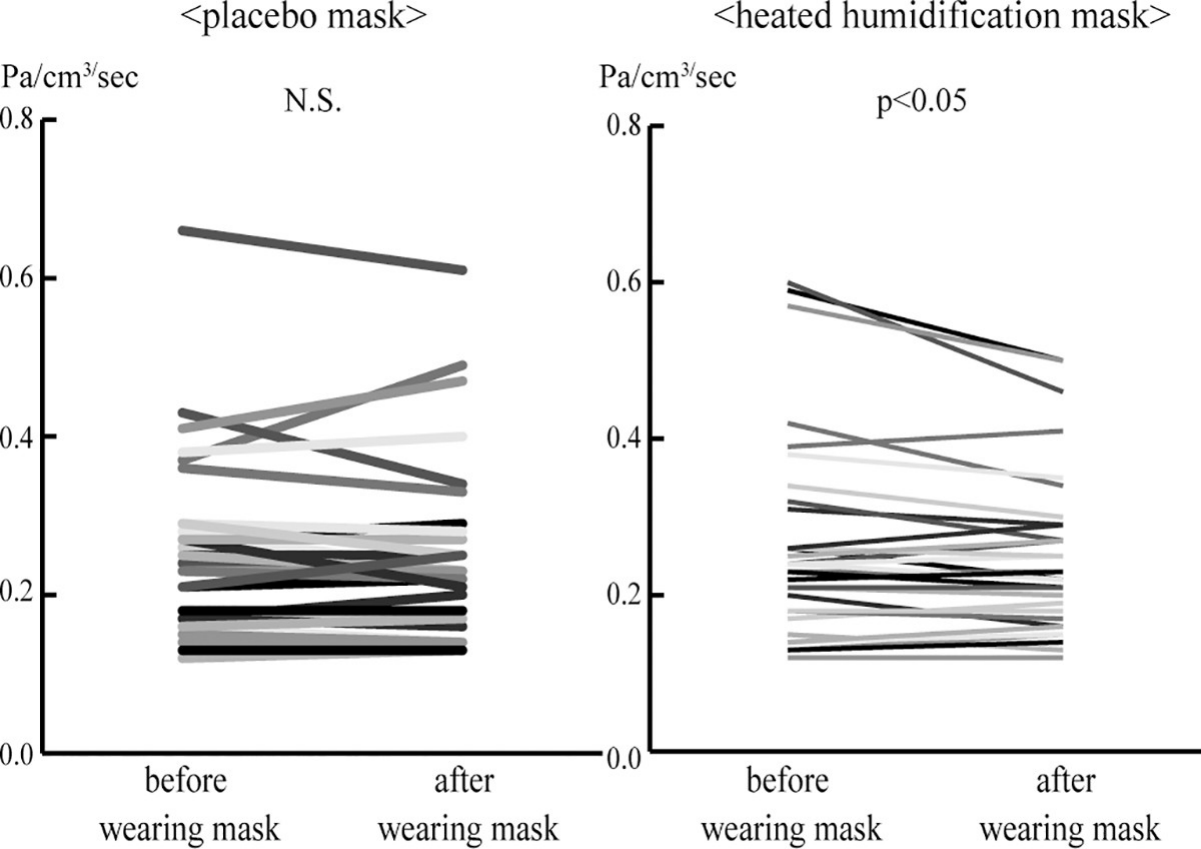
Nasal resistance before and after wearing placebo mask or heated humidification mask. Although placebo mask did not change the nasal resistance, heated humidification mask decreased nasal resistance.

Four subjects were excluded from the analysis due to measurement difficulty, because of unilateral complete nasal obstruction. Although the placebo mask did not change the nasal resistance before and after wearing mask, the heated humidification mask decreased nasal resistance (before vs. after; 0.25 ± 0.11: vs. 0.25 ± 0.11, N.S., 0.26 ± 0.13: vs. 0.25 ± 0.10, p<0.05, respectively) (Fig 3).

### Breathing pattern and Heart rate variability

**Fig 4:**
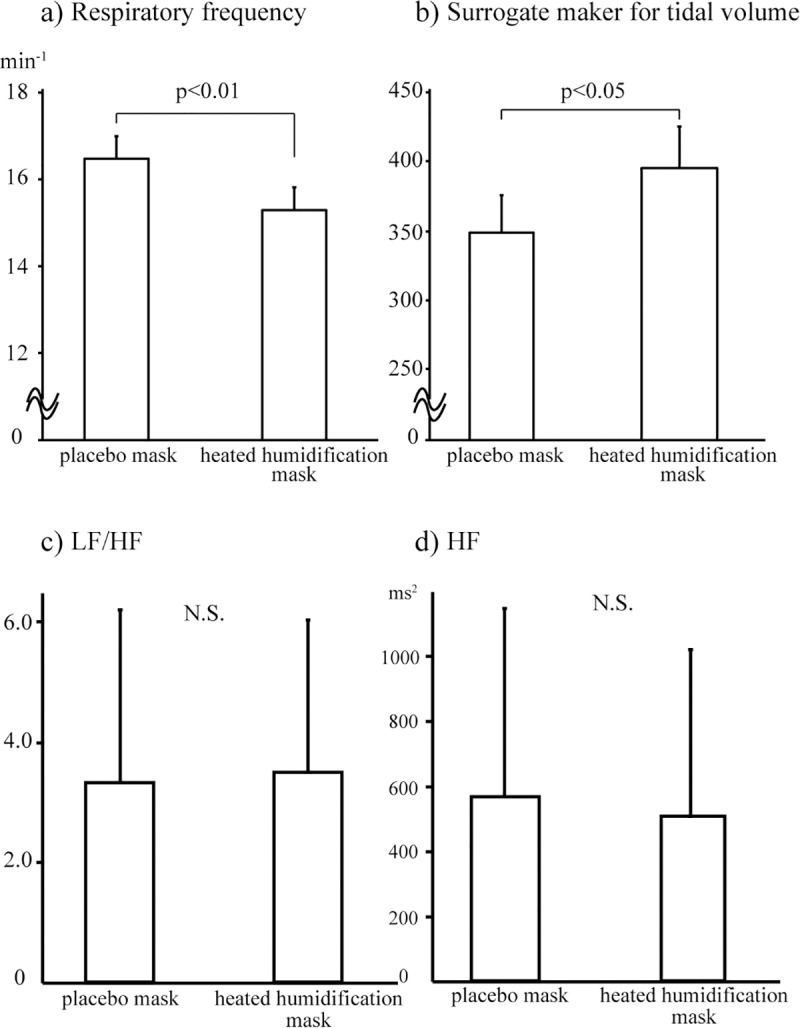
Breathing pattern and heart rate variability during wearing placebo mask or heated humidification mask. The heated humidification mask decreased respiratory frequency and simultaneously increased surrogate maker for tidal volume compared with placebo mask. LF/HF and HF were not significantly different between two masks.

The heated humidification mask decreased respiratory frequency and simultaneously increased the surrogate maker for tidal volume compared with placebo mask (Fig 4A and 4B). However, LF/HF and HF were not significantly different between two masks (Fig 4C and 4D).

## Discussion

In the current study, we demonstrated positive effects of our newly developed heated humidification mask. The heated humidification mask improved subjective feelings and decreased nasal resistance. More interestingly, the heated humidification mask modulated the breathing pattern, i.e., there appeared to be a decreased respiratory frequency accompanying simultaneous increases in surrogate tidal volume measures.

In general, expired air contains a lot of water vapor. Thus, the placebo mask has been considered to have a certain amount of moisturizing effect. Actually, the placebo mask improved subjective feelings of dry nose and dry throat in this study, but the effect was limited. Our newly developed heated humidification mask can provide more heat and humidity to nasopharynx and respiratory tract than placebo mask. The heated humidification mask also gave comfortable and relaxed feeling and reduced nasal resistance, while the placebo mask did not change mood and nasal resistance.

In the current study, we demonstrated that the heated humidification mask changed breathing pattern which was from rapid shallow breathing to slow deep breathing. Rapid shallow breathing is associated with increased ventilatory work load and/or reduction in respiratory muscle strength, and therefore could lead to respiratory muscle fatigue[12]. Thus, the heated humidification mask might be able to alleviate fatigue. Breathing is also affected by emotion. Former studies demonstrated that levels of individual anxiety affected respiratory rate[13]. Chronic anxiety was characterized by frequent sighs, and patients with panic disorder exhibited irregular breathing compared with healthy controls[14]. Moreover, unpleasant odors increased respiratory rate and eventually caused rapid shallow breathing, while pleasant odors induced deep breathing[15]. Relaxation effect obtained by the heated humidification mask we used in the current study might have produced slow deep breathing.

In the current study, we did not directly prove that heated humidification mask changed breathing route which was from mouth breathing to nasal breathing. Even if we see the participant’s mouth opened or closed, it would not necessarily provide a determination of breathing route. A specific device to identify breathing route would be ideal, however, it is beyond the scope of this study. For this issue, further study would be needed in the near future. The only thing we can emphasize based up on our findings is that the heated humidification mask improved nasal resistance and subjective feelings. Thus, considering the general fact, namely chronic nasal obstruction leads to mouth breathing, the heated humidification mask presumably facilitates nasal breathing. As for exact mechanisms of how heated humidification improved nasal resistance, we have not explored it in the current study. However former reports indicated that cold and dry air on nasal mucosa increases nasal resistance due to mucosal blood flux and the release of vasoactive amines and leukotrienes[16, 17]. On the other hand, heated humidification has been shown to prevent the increase in mucosal blood flux[18] and decrease in the level of nasal lavage pro-inflammatory cytokines[19]. Thus we assume these effects support our results, but further study to elucidate the underlying mechanisms of the novel mask we used in the current study is needed.

There are potential limitations to our study. First, we did not perform the calibration of ventilation for the nasal pressure sensor before measuring breathing pattern. Although we do not know absolute value of tidal volume, we adopted amplitude of nasal pressure sensor instead, which was considered as a surrogate marker of tidal volume. We do think our signal data were sufficient, however, to compare breathing pattern in the case of comparison of two different masks within the same subject. Second, contrary to the results of subjective feelings, the heated humidification mask did not improve autonomic nerve function assessed by heart rate variability. The reason for this discrepancy could not be elucidated in the current study. Use of heart rate variability indices remains not without some debate for assessing sympathetic nerve activity with concomitant with conflicting results. Other methods such as direct muscle sympathetic nerve activity (MSNA) might have been more suitable for our assessment.

## Conclusions

In conclusion, the heated humidification mask we newly developed could provide the beneficial effects for humans producing slow deep breathing and improving subjective feelings as well as nasal resistance. There is possibility that heated humidification mask leads mouth breathing toward nasal breathing.

## Supporting information

S1 File (DOCX)

## Abbreviations

HF: high-frequency component
HSG: heat-and-steam generator
LF: low-frequency component
SD: standard deviation
VAS: visual analogue scale
