# Supplementary material for: The effects of heated humidification to nasopharynx on nasal resistance and breathing pattern
Source: PLoS One. 2019 Feb 6;14(2):e0210957. doi: 10.1371/journal.pone.0210957 (PMC6364899; doi:10.1371/journal.pone.0210957)
Supplement: Supplementary file 1 [file pone.0210957.s001.docx]

Nasal resistance

No	p- before	p- after	H- before	H- after
1	0.18	0.18	0.22	0.23
2	0.38	0.40	0.38	0.35
3	0.41	0.47	0.57	0.50
4	0.21	0.25	0.21	0.21
5	0.16	0.17	0.14	0.16
6	0.17	0.16	0.14	0.16
7	0.29	0.25	0.34	0.30
9	0.36	0.33	0.42	0.34
10	0.13	0.13	0.13	0.14
11	0.29	0.28	0.24	0.22
12	0.15	0.14	0.12	0.12
13	0.66	0.61	0.60	0.46
14	0.27	0.27	0.25	0.27
15	0.27	0.21	0.26	0.29
16	0.17	0.18	0.18	0.18
17	0.23	0.22	0.18	0.17
18	0.21	0.22	0.23	0.21
20	0.16	0.14	0.14	0.15
21	0.23	0.23	0.18	0.18
22	0.43	0.34	0.32	0.27
23	0.25	0.23	0.21	0.20
26	0.25	0.25	0.31	0.29
27	0.26	0.23	0.26	0.25
28	0.26	0.22	0.23	0.23
29	0.39	0.95	0.59	0.50
30	0.28	0.23	0.24	0.25
32	0.14	0.13	0.13	0.15
33	0.24	0.23	0.24	0.27
34	0.12	0.13	0.15	0.13
35	0.17	0.20	0.20	0.16
36	0.18	0.17	0.17	0.19
37	0.37	0.49	0.39	0.41
38	0.27	0.29	0.26	0.22

p: placebo mask, H: heated humidification mask

Respiratory frequency

No	p- before	p- after	H- before	H- after
1	16.19	19.59	17.77	18.69
2	19.17	20.82	18.57	19.15
3	18.88	18.84	18.46	17.64
4	18.11	12.54	17.52	14.09
5	16.78	15.80	17.21	16.78
6	18.46	14.76	12.16	15.43
7	12.16	11.31	10.30	9.99
9	11.29	15.19	12.92	12.55
10	18.53	17.22	17.78	16.54
11	17.41	17.87	16.78	16.82
12	21.06	21.16	20.69	19.79
13	14.62	14.76	18.12	16.83
14	16.37	16.46	15.87	16.11
15	16.53	16.31	15.83	16.16
16	18.62	20.15	18.94	18.30
17	14.96	17.78	17.56	11.89
18	20.92	22.20	19.83	16.14
19	17.35	17.36	16.18	9.23
20	17.14	15.98	16.48	11.77
21	15.65	17.09	14.26	12.87
22	17.27	15.24	16.53	15.23
23	11.98	12.99	13.85	11.52
24	10.37	12.30	9.48	10.53
25	19.08	17.71	19.16	18.06
26	17.18	15.97	14.60	14.69
27	17.62	19.17	12.60	15.55
28	17.50	16.84	17.77	17.86
29	8.01	7.21	9.41	7.36
30	16.33	14.55	15.70	15.51
31	20.75	18.95	21.56	19.02
32	11.52	12.97	12.04	14.51
33	16.75	17.13	18.32	16.91
34	19.10	18.06	16.69	17.66
35	15.07	13.82	13.11	13.93
36	20.09	21.79	20.53	21.22
38	14.84	15.02	12.18	13.91

Surrogate maker for tidal volume

p- before	p- after	H- before	H- after
94.65	219.44	86.75	214.71
105.78	139.48	160.53	94.39
142.33	212.90	146.19	266.38
20.72	110.51	20.94	118.09
113.82	136.90	107.52	113.10
385.74	410.07	854.45	637.05
224.15	379.46	244.75	498.64
362.03	402.30	372.36	493.82
245.96	414.65	342.02	342.74
123.15	275.33	141.31	242.68
207.41	336.05	292.22	345.60
628.68	725.78	531.90	628.87
344.98	441.44	316.92	386.00
221.72	267.74	221.73	355.82
179.39	250.83	172.34	278.92
143.48	205.48	190.09	481.16
152.96	180.18	104.35	167.55
277.93	297.66	252.95	636.67
127.03	190.97	178.24	374.52
204.22	229.46	267.94	467.43
236.68	418.76	346.95	491.00
329.88	518.50	320.21	504.40
372.89	469.13	334.52	416.14
216.19	376.78	284.14	373.01
271.38	432.43	288.31	424.52
140.85	265.08	199.68	468.14
248.15	270.02	238.67	301.78
500.62	765.72	431.06	783.98
214.15	339.11	164.83	320.88
151.86	254.24	202.88	242.06
784.78	749.86	641.97	706.00
266.17	405.77	276.86	558.01
167.80	195.83	166.46	153.24
450.14	552.65	525.41	542.71
235.42	327.81	216.60	344.43
227.24	374.96	280.34	441.87

HF

p- before	p- after	H- before	H- after
133.75	134.33	104.73	152.40
76.71	31.33	71.87	42.81
169.11	146.41	275.83	292.88
724.92	682.10	746.89	367.44
217.77	366.01	411.73	467.83
188.97	342.63	558.62	236.59
176.20	254.87	217.08	315.08
695.78	691.25	962.28	1335.07
396.19	385.96	359.46	258.65
419.84	691.23	454.82	406.72
333.44	359.94	281.71	301.63
1113.62	735.62	543.13	959.79
323.63	339.45	446.23	385.60
1831.49	3114.23	712.73	1441.29
34.24	49.77	56.93	60.23
187.02	99.21	185.01	125.67
621.78	815.32	1007.67	604.48
468.73	1339.76	561.96	2483.62
469.46	949.34	477.93	486.99
430.17	514.43	599.47	471.55
321.76	488.39	259.23	346.45
272.14	389.97	153.37	467.37
119.43	1664.53	394.98	478.34
830.29	480.35	1106.83	996.98
282.84	383.81	149.18	168.39
133.66	144.49	151.81	139.93
72.47	198.54	232.97	203.37
524.19	436.12	491.07	349.80
342.40	437.43	110.43	257.46
1113.94	1454.77	1302.74	1259.80
291.43	510.34	258.43	335.25
73.72	76.84	112.39	69.26
553.49	594.04	722.99	846.51
140.39	196.70	206.57	185.62
450.18	456.30	503.84	590.94

LF/ HF

p- before	p- after	H- before	H- after
1.52	2.38	6.28	3.68
10.47	4.01	11.07	5.68
8.19	7.19	3.52	1.48
1.21	1.74	1.13	4.33
8.90	5.43	6.38	2.83
9.71	5.39	4.64	4.06
3.49	1.95	4.71	4.45
2.39	2.10	3.38	1.51
8.61	7.83	7.71	5.31
1.94	1.18	1.35	1.58
1.01	2.35	3.85	2.21
0.40	0.48	2.39	2.87
3.68	3.96	3.19	3.92
0.45	0.94	2.57	1.11
6.48	3.70	4.89	5.01
4.45	9.89	11.74	13.94
3.31	2.20	0.95	4.62
0.75	0.96	1.06	0.71
4.18	2.74	6.56	2.52
3.54	3.61	2.52	2.26
2.31	4.28	3.07	6.23
6.91	2.04	16.45	6.72
1.25	0.64	2.25	1.86
1.06	3.95	1.15	1.75
2.37	1.27	1.31	1.85
9.38	3.20	3.16	2.55
21.53	13.90	4.16	7.33
2.60	1.25	1.92	2.79
0.87	1.27	1.64	1.26
1.41	0.26	0.41	1.35
2.97	1.68	2.39	5.56
6.13	4.76	3.25	4.14
0.78	1.07	0.31	0.23
1.99	1.50	1.49	1.78
4.45	5.42	2.06	2.42
